# Prognostic value of soluble ST2 and soluble LR11 on mortality and cardiovascular events in peritoneal dialysis patients

**DOI:** 10.1186/s12882-020-01886-7

**Published:** 2020-06-15

**Authors:** Yu Bum Choi, Mi Jung Lee, Jung Tak Park, Seung Hyeok Han, Shin-Wook Kang, Tae-Hyun Yoo, Hyung Jong Kim

**Affiliations:** 1 Department of Internal Medicine, CHA Bundang Medical Center, CHA University, 59 Yatap-ro, Bundang-gu, Seongnam-si, Korea; 2 Department of Medicine, Graduate School of Yonsei University College of Medicine, Seoul, Korea; 3 Department of Internal Medicine, College of Medicine, Institute of Kidney Disease Research, Yonsei University, Seoul, Korea

**Keywords:** Biomarker, Major adverse cardiac and cerebrovascular events, Peritoneal dialysis, Soluble ST2, Soluble LR11

## Abstract

**Background:**

Although the soluble form of suppression of tumorigenicity 2 (sST2) and soluble low-density lipoprotein receptor relative with 11 ligand-binding repeats (sLR11) have emerged as novel cardiovascular biomarkers in patients with cardiovascular disease, their prognostic value has not been fully investigated in peritoneal dialysis (PD) patients.

**Methods:**

We included 74 prevalent PD patients from a prospective cohort and measured serum sST2 and sLR11 concentrations by an enzyme-linked immunosorbent assay. The association of these biomarkers and all-cause mortality and major adverse cardiac and cerebrovascular events (MACCEs) was evaluated.

**Results:**

During a follow-up of 38.5 months, all-cause deaths and MACCEs were observed in 13 (17.6%) patients and 23 (31.3%) patients. Multivariable Cox analyses demonstrated that greater sST2 was independently associated with higher risk of all-cause mortality (≥75.8 ng/mL; hazard ratio [HR] = 5.551; 95% confidence interval [CI] = 1.360–22.660) and MACCEs (≥72.5 ng/mL; HR = 4.609; 95% CI = 1.608–13.208). Furthermore, sST2 showed additive predictive value for mortality to the base model including traditional risk factors (net reclassification index = 0.598, *P* = 0.04). sLR11 was not significantly associated with all-cause mortality or MACCE.

**Conclusions:**

sST2, but not sLR11, indicated a significant prognostic value for all-cause mortality and cardiovascular events in PD patients. Further research is needed to validate emerging biomarkers in these populations.

## Background

Although the survival rate of patients with chronic kidney disease (CKD) has improved, cardiovascular disease remains a major cause of mortality in this population [1]. In CKD, cardiovascular changes are attributed to the kidney disease itself or to dialysis treatment when CKD progresses to end-stage renal disease (ESRD) [1, 2]. Indeed, approximately 20% or more of patients on dialysis develop systolic or diastolic dysfunction, suggesting that cardiovascular abnormalities are prevalent in ESRD patients [3]. Therefore, because predicting the risk of cardiovascular disease is expected to play a crucial role in improving the prognosis of cardiovascular disease, several studies have addressed this issue by evaluating new biomarkers.

Suppression of tumorigenicity 2 (ST2) is a member of the interleukin 1 receptor family, and its expression is stimulated by myocardial stress and injury [4]. ST2 has two different forms: a cardio-protective transmembrane receptor (ST2L) and a soluble decoy receptor (sST2) [5–7]. In contrast to ST2L, high concentrations of sST2 are associated with cardiac fibrosis, myocardial hypertrophy and ventricular dysfunction [6–8]. Recent evidence has indicated that sST2 is a useful prognostic marker, especially in congestive heart failure [9, 10]. Low-density lipoprotein receptor relative with 11 ligand-binding repeats (LR11), a member of the low-density lipoprotein receptor relatives, is highly expressed in vascular smooth muscle cells exhibiting enhanced migration, which leads to atherosclerosis [11–15]. In addition to LR11, the soluble form of LR11 (sLR11) also promotes vascular smooth muscle cell proliferation and migration, suggesting that sLR11 levels reflect the degree of vascular smooth muscle cell migration [14–21]. In patients with coronary artery disease [18, 19], familial hypercholesterolemia [21] and type 2 diabetes [16, 20], sLR11 levels were found to be increased.

To date, the prognostic value of sST2 and sLR11 has not been investigated in patients undergoing peritoneal dialysis (PD). Only a few studies have evaluated the association between clinical outcomes and sST2 in non-dialysis CKD patients and ESRD patients on haemodialysis (HD) or haemodiafiltration [22–25] and there are no studies that have focused on sLR11 in patients with kidney diseases. Therefore, we assessed the association of sST2 and sLR11 with adverse clinical outcomes in PD patients. Moreover, we compared the predictive value of sST2 and sLR11 with high-sensitivity C-reactive protein (hs-CRP) to test their clinical relevance as emerging cardiovascular biomarkers in these populations.

## Methods

### Study participants

This study involved participants from a prospective observational cohort of prevalent PD patients that was designed to investigate factors associated with cardiovascular risk and mortality in the Yonsei University Health System (YUHS). The details of this cohort were reported previously [26]. Among the 102 patients initially enrolled, 5 participants who had insufficient sample volumes for analysis, 7 participants who withdrew before additional blood analysis and 21 participants who declined to store their blood samples were excluded. Finally, 74 participants were analyzed in this study.

### Collection of clinical and biochemical data

Clinical data, including age, sex, cause of ESRD, duration of PD treatment, cardiovascular disease history, smoking status, and medications, were collected at study enrolment by a well-trained study coordinator. Smoking history was collected using a questionnaire. Smoking status was categorized as never-smokers and ever-smokers. Cause of ESRD, duration of PD treatment, comorbid diseases, and medications were collected by careful chart review. Cardiovascular disease was defined as coronary artery disease, peripheral artery disease, or cerebrovascular disease. Coronary artery disease was defined as an acute coronary syndrome or angina requiring coronary artery angioplasty or coronary artery bypass. Peripheral artery disease was defined as ischaemic limb loss and/or ulceration requiring peripheral artery revascularization. Cerebrovascular disease was defined as a transient ischaemic attack, cerebral infarction or carotid endarterectomy. Diabetes mellitus was defined as a history of diagnosis or taking oral hypoglycaemic agents or insulin treatment. Anthropometric measures and blood pressure were determined by a single skilled nurse from our PD unit in the morning after complete overnight dialysate emptying. Height and weight were measured and body mass index was calculated. Blood pressure was measured using a validated automatic oscillometric device. The average value of two measures was recorded. To alleviate the variability from overnight peritoneal dialysate, overnight dialysate was not changed. Overnight fasting blood (12-h) was collected at the time of study enrolment and biochemical variables were measured in the YUHS laboratory. For storing blood samples, an additional 10 mL of whole blood was collected, and the aliquots of serum and plasma were stored in a deep freezer at − 70 °C. Kt/V urea was calculated by PD Adequest 2.0 for Windows software (Baxter Healthcare, Deerfield, IL, USA) for the assessment of dialysis adequacy.

### Assessment of sST2, sLR11, and hs-CRP concentrations

Using stored samples, serum sST2, and sLR11 concentrations were measured by a colourimetric enzyme-linked immunosorbent assay (ELISA; sST2, Presage® ST2 Assay, Critical Diagnostics, San Diego, CA, USA; sLR11, MBS-167426, MyBioSource, San Diego, CA, USA). Inter- and intra-assay coefficients of variation were both < 10% for sST2, and < 15, and < 10%, respectively, for sLR11. hs-CRP concentrations were measured by a BNII analyser (Dade Behring, Newark, DE, USA) using a latex-enhanced immunonephelometric method.

### Follow-up and study outcomes

Patients were followed every 3 months at the PD unit of the YUHS through September 2018. All adverse clinical events, including deaths and hospitalizations, were recorded in the database of our PD unit. The primary outcome was all-cause mortality and the secondary outcome was a major adverse cardiac and cerebrovascular event (MACCE). Death or hospitalization from an acute coronary syndrome, stable angina performing coronary revascularization procedures, newly developed congestive heart failure or cerebrovascular events was defined as MACCE. Patients who did not experience primary outcome were censored at the end of the study date and those who were lost to follow-up were censored at the date of the last examination. If patients underwent a kidney allograft or changed dialysis modality to HD, they were censored at the date of the last PD treatment. If death occurred within 2 months after transfer to HD, the death was considered as a death from PD.

### Statistical analysis

Statistical analysis was performed using SPSS for Windows version 20.0 (IBM Corp., Armonk, NY, USA) and R software (R Foundation for Statistical Computing, Vienna, Austria; www.r-project.org). Continuous variables are expressed as the mean ± standard deviation (SD) or the median (interquartile range) and categorical variables are expressed as the raw number (percentage). To evaluate the association of sST2 and sLR11 with study outcomes, study participants were dichotomized based on the median values of each marker. The cut-off value for sST2 and sLR11 was also calculated from receiver operating characteristic (ROC) analyses with the calculated area under the ROC curve (AUC) in accordance with previous studies [23, 24]. The value showing the highest sensitivity was determined as the cut-off. The calculated cut-off values of sST2 for all-cause mortality and MACCEs were 75.8 ng/mL (sensitivity, 69.2%; specificity, 60.7%) and 72.5 ng/mL (sensitivity, 65.2%; specificity, 58.8%). The calculated cut-off of sLR11 for all-cause mortality and MACCEs was 14.9 ng/mL (61.5% sensitivity and 44.3% specificity for all-cause mortality; 52.5% sensitivity and 41.2% specificity for MACCEs). Cumulative survival curves were constructed using the Kaplan–Meier method and between-group survival was compared using the log-rank test. To determine the independent prognostic value of sST2 and sLR11 for all-cause mortality and MACCEs, Cox proportional regression analyses were performed. A multivariable model was constructed with significant variables from univariate analyses: sex, age, diabetes mellitus, and cardiovascular disease. Subsequent multivariable analysis was performed with additional variables that were known to be associated with adverse clinical outcomes in PD patients: duration of PD, smoking status, body mass index, and haemoglobin concentrations. Using a fractional polynomial model, sST2 and sLR11 were also evaluated as continuous variables. To compare the predictive value of sST2, sLR11, and hs-CRP for all-cause mortality and MACCEs, we evaluated the additive effects of each biomarker on the base model: model 1, base model + sST2; model 2, base model + sLR11; model 3, base model + hs-CRP. The continuous net reclassification index (NRI), the integrated discrimination improvement (IDI) and *c* Statistics were calculated to ascertain which biomarker improved prediction of the primary outcome when added to the base model. A *P* value of less than 0.05 was considered to indicate statistical significance.

## Results

### Baseline characteristics of participants

The baseline characteristics of study participants are shown in Table 1. The mean age of patients was 53.9 ± 11.8 years, and 47 patients (63.5%) were men. The median PD duration was 30.0 (16.0–96.0) months. A total of 21 patients (28.4%) were diabetic and 10 patients (13.5%) had a history of cardiovascular disease. Diabetic kidney disease was the most common cause of ESRD, followed by hypertensive nephrosclerosis and glomerulonephritis. The mean concentrations of serum sST2 and sLR11 were 75.0 ± 26.6 ng/mL and 16.4 ± 4.8 ng/mL, respectively, the median value of hs-CRP was 0.96 (0.62–2.10) mg/L.

**Table 1:** Baseline characteristics of study participants

	*N* = 74
Age (years)	53.9 ± 11.8
Male, *n* (%)	47 (63.5)
Duration of PD (months)	30.0 (16.0–96.0)
Cases of ESRD, *n* (%)	
Diabetic kidney disease	21 (28.4%)
Hypertensive nephrosclerosis	19 (25.7%)
Glomerulonephritis	17 (23.0%)
Polycystic kidney disease	3 (4.1%)
^a^Others	3 (4.1%)
Unknown	11 (14.9%)
Diabetes mellitus, *n* (%)	21 (28.4)
Cardiovascular disease, *n* (%)	10 (13.5)
Ever-smokers, *n* (%)	29 (39.2)
Lipid-lowering therapy, *n* (%)	28 (37.8)
Systolic blood pressure (mmHg)	133.7 ± 21.5
Diastolic blood pressure (mmHg)	75.0 ± 11.6
Body mass index (kg/m^2^)	23.1 ± 2.8
Hemoglobin (g/L)	104 ± 11
Glucose (mmol/L)	5.3 ± 2.4
Blood urea nitrogen (mmol/L)	7.4 ± 2.1
Creatinine (μmol/L)	972.4 ± 371.3
Albumin (g/L)	35 ± 5
Triglyceride (mmol/L)	1.3 ± 0.8
Total cholesterol (mmol/L)	4.3 ± 0.9
LDL cholesterol (mmol/L)	2.4 ± 0.7
HDL cholesterol (mmol/L)	1.1 ± 0.3
Calcium (mmol/L)	2.2 ± 0.2
Phosphorous (mmol/L)	1.7 ± 0.5
Total Kt/V urea (per week)	2.1 ± 0.4
nPCR (g/kg/day)	0.97 ± 0.17
hs-CRP (mg/L)	0.96 (0.62–2.10)
sST2 (ng/mL)	75.0 ± 26.6
sLR11 (ng/mL)	16.4 ± 4.8

Data are expressed as mean ± standard deviation, median (interquartile range), or number of patients (percent)

*Abbreviations*: *hs-CRP* high-sensitivity C-reactive protein, *HDL* high-density lipoprotein, *Kt/V urea* fractional urea clearance, *LDL* low-density lipoprotein, *nPCR* normalized protein catabolic rate, *PD* peritoneal dialysis, *sLR11* soluble form of low-density lipoprotein receptor relative with 11 ligand-binding repeats, *sST2* soluble form of suppression of tumorigenicity 2

^a^Others: interstitial nephritis, obstructive uropathy, or post status of nephrectomy

### Prognostic value of sST2 for all-cause mortality and MACCE

**Fig. 1:**
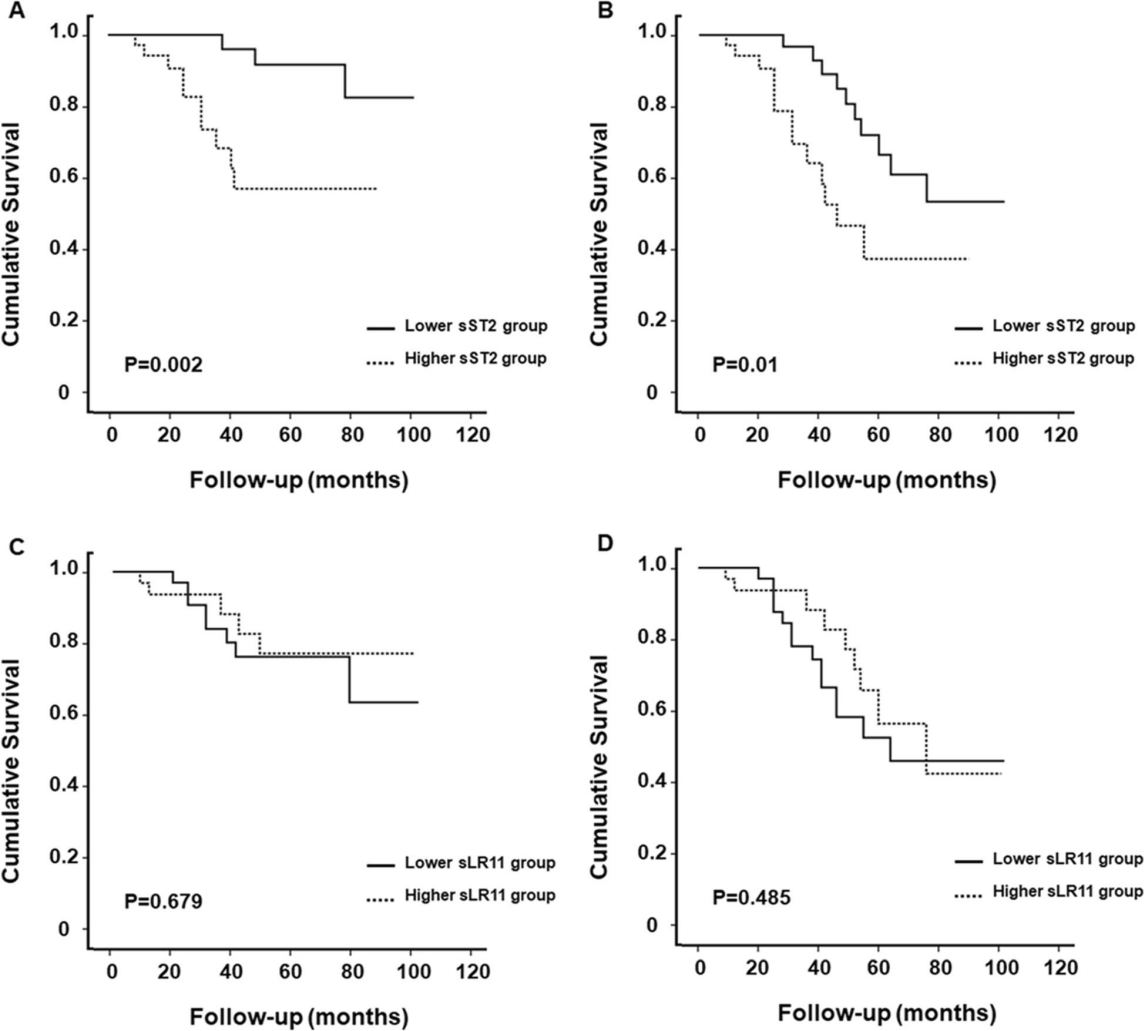
Kaplan-Meier plots for study outcomes for high and low sST2 and sLR11 groups. The high sST2 group had significantly greater risk of all-cause death (**a**) and MACCEs (**b**) compared with the low sST2 group. There were no significant differences in the risk of all-cause death (**c**) and MACCEs (**d**) between the high and low sLR11 groups. *Abbreviations*: MACCE, major adverse cardiac and cerebrovascular event; sLR11, soluble form of low-density lipoprotein receptor relative with 11 ligand-binding repeats; sST2, soluble form of suppression of tumorigenicity 2

**Fig. 2:**
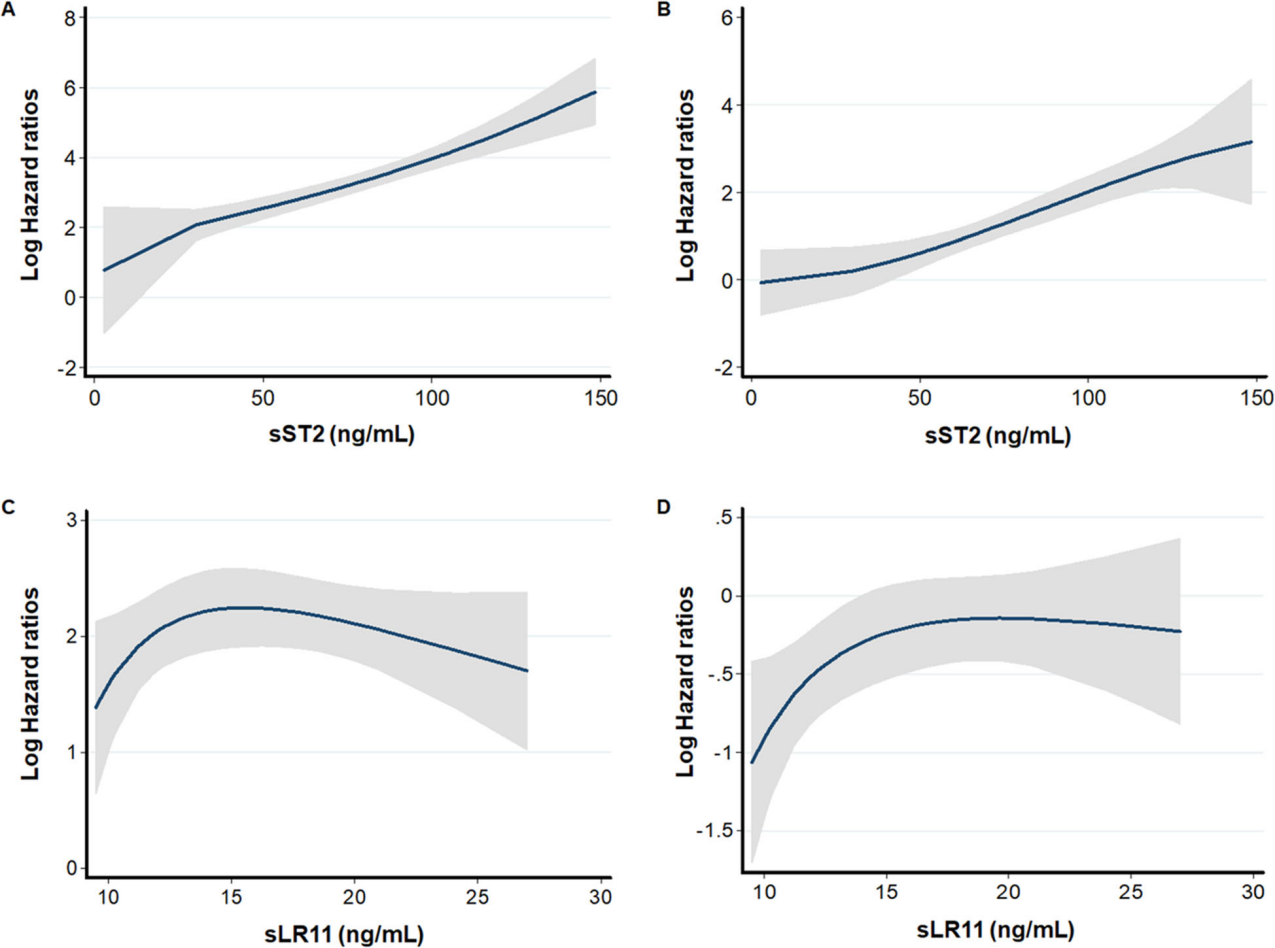
Multivariable fractional polynomial graph for association of sST2 and sLR11 with study outcomes. Hazard ratios were calculated after adjustment for age, sex, peritoneal dialysis duration, diabetes mellitus, history of cardiovascular disease, smoking status, body mass index, and hemoglobin for all-cause death and MACCE. sST2 indicated a linear relationship with the risk of (**a**) all-cause mortality and (**b**) MACCEs. sLR11 did not show a remarkable trend with (**c**) all-cause mortality and (**d**) MACCEs. Shaded areas indicate 95% confidence intervals. *Abbreviations*: MACCE, major adverse cardiac and cerebrovascular event; sLR11, soluble form of low-density lipoprotein receptor relative with 11 ligand-binding repeats; sST2, soluble form of suppression of tumorigenicity 2

During a median follow-up of 38.5 months, 13 (17.6%) patients died and there were 23 (31.3%) MACCEs. When patients were dichotomized based on the median value of sST2 (70.9 ng/mL), crude all-cause death rates were 1.74 and 10.18 per 100 person-years, and MACCE rates were 6.15 and 13.47 per 100 person-years in the low and high sST2 groups, respectively (Table S1). The cumulative survival rate was significantly lower in the high sST2 group (log-rank test; *P* = 0.002 for all-cause mortality and *P* = 0.01 for MACCEs) (Fig. 1). In multivariable Cox regression analysis, the high sST2 (above the median) was significantly associated with increased risk for all-cause mortality and MACCEs (Table 2). When patients were categorized using calculated cut-off values of sST2, the high sST2 group had a 5.551- and 4.609-fold increased risk for all-cause mortality and MACCEs compared to the lower sST2 group. Moreover, the prognostic value of sST2 remained significant as a continuous variable (per 1 SD increase, hazard ratio [HR] = 1.943; 95% confidence interval [CI] = 1.124–3.359 for all-cause mortality and HR = 1.630; 95% CI = 1.073–2.477 for MACCEs). When dialysis adequacy was added to multivariable models, the prognostic value of sST2 remained consistent (Table S2). Multivariable fractional polynomial analysis indicated that the risk of all-cause mortality and MACCEs increased steadily with higher sST2 levels, showing a linear relationship (Fig. 2).

**Table 2:** Multivariable Cox proportional hazard models of sST2 and sLR11 for all-cause mortality and MACCEs

	Crude HR (95% CI)	^a^Adjusted HR 1 (95% CI)	^b^Adjusted HR 2 (95% CI)
**All-cause mortality**			
**sST2**			
^c^sST2 ≥ 70.9 ng/mL (vs. < 70.9)	6.715 (1.749–25.784)	9.638 (2.180–42.602)	10.144 (2.156–47.734)
^d^sST2 ≥ cut-off (vs. <cut-off)	9.459 (2.034–43.988)	12.189 (2.408–61.696)	5.551 (1.360–22.660)
sST2 (per 1 SD increase)	2.179 (1.351–3.515)	1.921 (1.151–3.207)	1.943 (1.124–3.359)
**sLR11**			
^c^sLR11 ≥ 15.2 ng/mL (vs. < 15.2)	0.791 (0.258–2.422)	0.630 (0.187–2.130)	0.601 (0.168–2.145)
^e^sLR11 ≥ cut-off (vs. <cut-off)	1.271 (0.415–3.896)	0.944 (0.271–3.286)	0.859 (0.211–3.503)
sLR11 (per 1 SD increase)	0.884 (0.503–1.555)	0.763 (0.397–1.467)	0.719 (0.356–1.451)
**MACCEs**			
**sST2**			
^c^sST2 ≥ 70.9 ng/mL (vs. < 70.9)	2.056 (1.259–6.940)	4.623 (1.743–12.263)	3.928 (1.413–10.918)
^d^sST2 ≥ cut-off (vs. <cut-off)	3.776 (1.546–9.218)	5.445 (2.020–14.679)	4.609 (1.608–13.208)
sST2 (per 1 SD increase)	1.748 (1.179–2.592)	1.683 (1.120–2.529)	1.630 (1.073–2.477)
**sLR11**			
^c^sLR11 ≥ 15.2 ng/mL (vs. < 15.2)	0.743 (0.321–1.723)	0.736 (0.29301.849)	0.737 (0.271–2.002)
^e^sLR11 ≥ cut-off (vs. <cut-off)	1.278 (0.551–2.965)	1.315 (0.532–3.250)	1.315 (0.487–3.548)
sLR11 (per 1 SD increase)	0.951 (0.634–1.428)	0.947 (0.606–1.481)	0.927 (0.577–1.490)

*Abbreviations*: *CI* confidence interval, *HR* hazard ratio, *MACCE* major adverse cardiac and cerebrovascular event, *PD* peritoneal dialysis, *SD* standard deviation, *sLR11* soluble form of low-density lipoprotein receptor relative with 11 ligand-binding repeats, *sST2* soluble form of suppression of tumorigenicity 2

^a^Adjusted HR 1 was calculated after adjustment of age, sex, diabetes mellitus, and history of cardiovascular disease

^b^Adjusted HR 2 was calculated after adjustment of age, sex, PD duration, diabetes mellitus, history of cardiovascular disease, smoking status, body mass index, and hemoglobin concentrations

^c^Patients were dichotomized based on median values of sST2 (70.9 ng/mL) and sLR11 (15.2 ng/mL), respectively

^d^Patients were categorized into two groups based on the calculated cut-off of sST2 for all-cause mortality (75.8 ng/mL) and MACCEs (72.5 ng/mL)

^e^Patients were categorized into two groups based on the calculated cut-off of sLR11 (14.9 ng/mL) for all-cause mortality and MACCEs

### Prognostic value of sLR11 for all-cause mortality and MACCEs

Patients were dichotomized based on the median value of sLR11 (15.2 ng/mL). All-cause death rates were 5.34 and 4.12 per 100 person-years, and MACCE rates were 9.78 and 7.76 per 100 person-years in the low and high sLR11 groups, respectively (Table S1). There were no differences in all-cause death or MACCE-free survival rates between the high and low sLR11 groups (both *P* > 0.05) (Fig. 1). Multivariable Cox regression analysis showed there was no significant association between sLR11 and all-cause mortality or MACCEs, regardless of using median value (15.2 ng/mL) or calculated cut-off (14.9 ng/mL) (Table 2). Fractional polynomial analysis indicated a non-linear relationship (Fig. 2).

### Predictive value of sST2, sLR11, and hs-CRP for all-cause mortality and MACCE

**Fig. 3:**
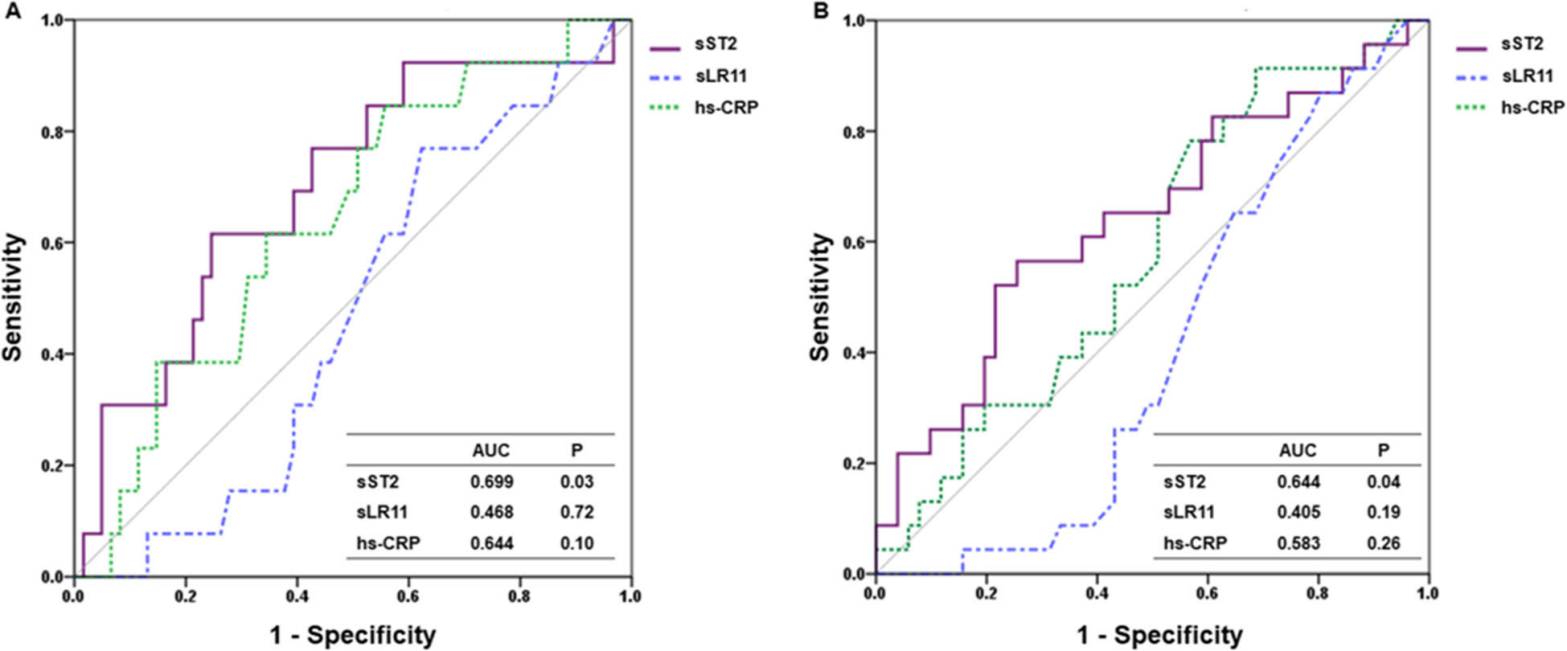
Receiver operating characteristic curves for sST2, sLR11, and hs-CRP to predict all-cause mortality and MACCEs. sST2 exhibited a significant predictive value for (**a**) all-cause mortality and (**b**) MACCEs (both *P* < 0.05). *Abbreviations*: hs-CRP, high-sensitivity C-reactive protein; MACCE, major adverse cardiac and cerebrovascular event; sLR11, soluble form of low-density lipoprotein receptor relative with 11 ligand-binding repeats; sST2, soluble form of suppression of tumorigenicity 2

The predictive value of sST2, sLR11, and hs-CRP was evaluated using ROC analysis (Fig. 3), continuous NRI, IDI, and *c* Statistics (Table 3). The AUCs of sST2 for all-cause mortality and MACCEs were 0.699 (*P* = 0.03) and 0.644 (*P* = 0.04), respectively. In contrast, the AUC of sLR11 and hs-CRP were not significant. Furthermore, when sST2 was added to the base model that included traditional risk factors (model 1), only sST2 significantly improved the predictive ability of all-cause mortality (NRI = 0.598, P = 0.04). However, sLR11 (model 2) or hs-CRP (model 3) did not increase predictability for all-cause mortality. In terms of MACCE, there was no significant advantage from adding sST2, sLR11, or hs-CRP to the base model. Similar to IDI, the ability of discrimination was not significant in *c* Statistics*.*

**Table 3:** NRIs, IDIs, and *c* Statistics for prediction of all-cause mortality and MACCE

Models	NRI	^a^P	IDI	^a^P	*c* Statistics	^a^P
	(95% CI)		(95% CI)			
**All-cause mortality**						
Base model	Reference		Reference		0.729	
Model 1	0.598 (0.039 to 1.156)	0.04	0.029 (− 0.0068 to 0.0655)	0.11	0.863	0.48
Model 2	0.225 (− 0.372 to 0.821)	0.46	0.005 (−0.014 to 0.024)	0.61	0.731	0.19
Model 3	0.477 (−0.106 to 1.059)	0.11	0.027 (−0.0005 to 0.060)	0.10	0.765	0.71
**MACCE**						
Base model	Reference		Reference		0.718	
Model 1	0.385 (−0.099 to 0.870)	0.12	0.008 (−0.014 to 0.030)	0.49	0.801	0.46
Model 2	0.133 (−0.357 to 0.627)	0.59	0.019 (−0.013 to 0.050)	0.24	0.705	0.33
Model 3	0.007 (−0.481 to 0.495)	0.9	0.001 (−0.002 to 0.007)	0.82	0.720	0.47

Base model: adjusted for age, sex, PD duration, smoking, diabetes mellitus, cardiovascular disease, body mass index, and hemoglobin

Model 1: base model + sST2

Model 2: base model + sLR11

Model 3: base model + hs-CRP

*Abbreviations*: *CI* confidence interval, *IDI* integrated discrimination improvement, *hs-CRP* high-sensitivity C-reactive protein, *PD* peritoneal dialysis, *MACCE* major adverse cardiac and cerebrovascular event, *NRI* net reclassification index, *sLR11* soluble form of low-density lipoprotein receptor relative with 11 ligand-binding repeats, *sST2* soluble form of suppression of tumorigenicity 2

^a^vs. Base model

## Discussion

In the present study, we first demonstrated that higher sST2 concentrations are independently associated with a greater risk of all-cause mortality and MACCEs in prevalent PD patients. In contrast, we did not find a significant association between sLR11 and study outcomes. Only sST2 improved the predictive ability of all-cause mortality, when added to the base model including traditional risk factors.

Previous studies indicated that ST2L, the transmembrane receptor form of ST2, exhibited cardio-protective effects associated with binding to interleukin-33 (IL-33), which results in a reduction in cardiomyocyte apoptosis and fibrosis or hypertrophy of injured cardiac tissues [4, 5]. The soluble form of ST2, sST2, is a decoy receptor [6, 7]. As its concentration increases, sST2 competitively binds to IL-33 and disrupts the ST2L/IL-33 interaction, thus inhibiting cardio-protective signalling [6, 7]. Therefore, the sST2 level has been widely investigated as a biomarker of cardiovascular disease, especially in patients with congestive heart failure [8–10]. Two recent meta-analyses demonstrate clearly that sST2 has a significant prognostic value in heart failure patients [9, 10]. In chronic heart failure, sST2 also showed a significant prognostic value for all-cause and cardiovascular mortality [9]. A study of acute congestive heart failure found that higher sST2 concentrations at both admission and discharge are significantly associated with all-cause and cardiovascular mortality or hospitalization due to heart failure [10]. Furthermore, repeated measurements of sST2 concentration are useful for assessing the clinical course of heart failure, suggesting that sST2 is a suitable biomarker for disease monitoring as well as prognosis [27].

Only a few studies have investigated the prognostic value of sST2 in patients with kidney diseases. One study involving 883 pre-dialysis CKD patients showed that higher sST2 concentrations are associated with increased mortality risk [25]. In a subgroup analysis of heart failure patients, high sST2 level remained a significant prognostic factor in patients with an estimated glomerular filtration rate < 60 mL/min/1.73 m^2^ [28]. In HD patients, two studies reported that sST2 had a significant association with all-cause mortality [23] and cardiovascular events [24]. In the current study, we revealed for the first time that prevalent PD patients with elevated sST2 levels have a greater risk of all-cause mortality and MACCEs. The significant prognostic value of sST2 remained unaltered when patients were categorized using both median value and calculated cut-off. To date, there is no definite sST2 cut-off for the prediction of clinical outcomes in populations with kidney diseases. Previous HD studies suggested a 48 ng/mL for all-cause mortality [23] or 58 ng/mL for MACCE [24], respectively. In this study, the calculated cut-off for sST2 was 75.8 ng/mL for all-cause mortality and 72.5 ng/mL for MACCE, which were higher than those of previous studies. In prevalent HD patients in Slovenia, the median value of sST2 was 28 ng/mL [23]. In a study of Korean incident HD patients, the median value of sST2 was 59.5 ng/mL [24]. Because our patients were prevalent PD patients with a median dialysis vintage of 30 months, we assumed that our patients may be at higher cardiovascular risk than incident dialysis patients, leading to higher cut-off and median value of sST2. However, we did not clarify the exact reason for the discrepancy in cut-off and median values of sST2, such as ethnicity, dialysis modality (HD or PD), or dialysis vintage. Future studies are needed to address this issue.

In the present study, the AUCs of sST2 for all-cause mortality and MACCE were significant and sST2 significantly improved the predictive ability for all-cause mortality when it was added to the base model that included traditional risk factors. From these results, we surmised that sST2 could be helpful in predicting adverse clinical outcomes in PD patients. However, the absolute values of the AUCs were not high (0.699 for all-cause mortality and 0.644 for MACCE) and sST2 did not show additive predictive value for MACCE compared with the base model. The discrimination index was not significant. These findings suggest that sST2 alone does not outweigh the predictive ability of traditional risk factors for cardiovascular outcomes. Nevertheless, fractional polynomial analyses found a linear association between sST2 concentrations and study outcome, which can be easily interpreted in clinical practice. Moreover, the sST2 concentration is independent of kidney function and CKD stage [28, 29] and not affected by haemodiafiltration procedures [30]. Interpretation of serum concentrations of biomarkers that are affected by kidney function or dialysis is complex and thus not easily applicable to real-world clinical settings. Based on these results, we speculate that sST2 can be a clinically relevant biomarker in PD patients. However, further investigation with a larger number of participants is necessary to evaluate the additive predictive value of sST2 for adverse cardiovascular outcomes in a PD population.

Previous studies have reported that sLR11 is implicated in coronary artery disease [18, 19], carotid atherosclerosis [20] and vascular calcification [21]. However, to date, sLR11 has not been investigated in patients with kidney diseases, and this study revealed that sLR11 concentration is not correlated with the risk of death or cardiovascular disease in prevalent PD patients. The mechanism by which LR11 mediates atherosclerosis reportedly involves the enhancement of vascular smooth muscle cell proliferation and migration [11–15]. LR11 produced by intimal smooth muscle cells induces increased smooth muscle cell migration in vitro via the upregulation of urokinase-type plasminogen activator receptor expression [15]. Of note, postprandial triglyceride-rich lipoproteins stimulate LR11 release from aortic smooth muscle cells, and overexpression of LR11 increases binding of triglyceride-rich lipoproteins but not low-density lipoprotein [31], suggesting a close association with triglycerides. In CKD patients, non-traditional risk factors, such as uraemia, inflammation, malnutrition or mineral-bone disease also contribute to a high risk of developing cardiovascular disease, in addition to the traditional risk factors such as advanced age, smoking, diabetes, and dyslipidaemia [32]. In this study, the mean triglyceride concentrations were 1.3 mmol/L, which is lower than the cut-off for hypertriglyceridemia (> 2.3 mmol/L) in the general Korean population. Triglyceride level was not a significant risk factor for mortality or MACCEs in the univariate Cox analysis (data not shown). Considered collectively, these data suggest that the lower contribution of dyslipidaemia to the development of cardiovascular disease in a CKD setting could explain the non-significant association between sLR11 levels and prognosis in our patients. Furthermore, uraemia and impaired kidney function can alter the expression or metabolism of sLR11 in patients with kidney diseases, and dialysis procedures can also affect sLR11 concentrations. However, there are no available data on sLR11 concentrations in ESRD patients, thus further studies are needed to clarify this issue.

This study has several limitations. First, the number of study participants was small, resulting in a small number of primary outcomes. Although our multivariable models were well fitted (Table S3), the potential risk of overfitting still exists. In addition, we cannot exclude type II errors for the non-significant prognostic value of sLR11. To mitigate these confounding effects, a fractional polynomial analysis was performed to explore the association of sST2 and sLR11 with primary outcome using continuous terms. In the fractional polynomial analysis, sST2 showed a linear relationship but sLR11 showed a non-linear relationship with study outcomes. Therefore, even if our study was underpowered to detect a significant difference, the clinical utility of sLR11 as a biomarker is low due to the complexity of its interpretation, at least in PD patients. However, our findings should be interpreted with caution and should be confirmed by external validation in another independent cohort. Furthermore, a large-scale prospective investigation is mandatory to confirm our results. Second, this study analyzed only Korean prevalent PD patients. We have presumed that our participants were at high risk of cardiovascular disease due to a relatively long duration of PD. However, on the contrary, our participants have been tolerable to PD treatment until study enrolment. Given the high mortality risk in the early period of dialysis commencement, investigating prevalent PD patients may lead to a survival bias. Third, in the current study, the proportion of participants who had diabetes mellitus was 28.4%, which is lower than the 50.2% of Korean dialysis patients [33]. Moreover, only 13.5% of the participants had a history of cardiovascular disease. These findings suggest that our study participants were more likely to be in a better clinical condition compared with other PD cohorts, introducing a selection bias. Furthermore, several patients were excluded from the original cohort due to lack of blood samples or withdrawal from further study participation, leading to the potential for another selection bias. To abrogate the effect of selection bias, we analyzed differences in baseline characteristics between patients with and without biomarker measurements. We found no significant differences in baseline characteristics except for PD duration and haemoglobin between patients who had available sST2 and sLR11 concentration measurements and those who did not (Table S4). Nevertheless, 74 patients may not be representative of all participants in our original cohort. Fourth, this study included a small number of participants from a single tertiary hospital in Korea. Because there are huge differences in medical resources and dialysis practice pattern among institutions, our results may not be generalized to other populations. Fifth, due to the observational nature of this study, we cannot evaluate the causal relationship between sST2 or sLR11 and the development of our primary outcome. Although we surmised that a significant association of sST2 with cardiac fibrosis, myocardial hypertrophy, or ventricular dysfunction observed in previous studies may contribute to the prognostic value of sST2 on mortality and MACCEs, underlying mechanisms were not investigated in this study. Furthermore, the effects of changes in sST2 and sLR11 on mortality or cardiovascular disease were not evaluated, exerting an unmeasured confounding effect. Although multivariable Cox models were constructed with significant variables from univariate analysis and additional variables which were known to be associated with adverse clinical outcome in PD patients, we cannot totally exclude residual confounding effects derived from the observational study. Sixth, because there is no definite cut-off for sST2 and sLR11 in patients with kidney diseases, we calculated the cut-off value using ROC analysis. These cut-offs need to be validated in further studies. Lastly, we only determined concentrations of sST2 and sLR11 and did not evaluate other emerging cardiovascular biomarkers, such as growth differentiation factor 15 or galectin 3. Determining and comparing the prognostic value of various new biomarkers is worthwhile. Despite these limitations, we believe that the current study provides useful information regarding the prognostic value of emerging cardiovascular biomarkers in PD patients.

## Conclusions

The present study reveals that sST2 is a significant prognostic factor in PD patients. However, we found no significant association between SLR11 and the study outcomes. These findings suggest that sST2 may be a useful biomarker for stratifying mortality and cardiovascular risk in patients undergoing PD. However, additional studies to validate these emerging cardiovascular biomarkers in this population are needed.

## Supplementary information

**Additional file 1: Table S1**. All-cause death and MACCE according to the sST2 and sLR11 groups. **Table S2.** Multivariable Cox proportional hazard models of sST2 and sLR11 for all-cause mortality and MACCEs with adjustment of dialysis adequacy. **Table S3.** Hosmer-Lemeshow test for calibration of models. **Table S4.** Baseline characteristics of patients with and without sST2 and sLR11 values.

## Data Availability

The datasets used for the current study are available from the corresponding author on reasonable request.

## Abbreviations

CKD: Chronic kidney disease
ESRD: End-stage renal disease
HD: Hemodialysis
hs-CRP: High-sensitivity C-reactive protein
IDI: Integrated discrimination improvement
MACCE: Major adverse cardiac and cerebrovascular event
NRI: Net reclassification index
PD: Peritoneal dialysis
sLR11: Soluble form of low-density lipoprotein receptor relative with 11 ligand-binding repeats
sST2: Soluble form of suppression of tumorigenicity 2
