# Supplementary material for: Prognostic value of soluble ST2 and soluble LR11 on mortality and cardiovascular events in peritoneal dialysis patients
Source: BMC Nephrol. 2020 Jun 15;21:228. doi: 10.1186/s12882-020-01886-7 (PMC7296670; doi:10.1186/s12882-020-01886-7)
Supplement: Supplementary file 1 — Additional file 1: Table S1. All-cause death and MACCE according to the sST2 and sLR11 groups. Table S2. Multivariable Cox proportional hazard models of sST2 and sLR11 for all-cause mortality and MACCEs with adjustment of dialysis adequacy. Table S3. Hosmer-Lemeshow test for calibration of models. Table S4. Baseline characteristics of patients with and without sST2 and sLR11 values. [file 12882_2020_1886_MOESM1_ESM.pdf]

Table S1. All-cause death and MACCE according to the sST2 and sLR11 groups

	All-cause death		MACCE	
	Events	Event rate (per 100 PY)	Events	Event rate (per 100 PY)
[a]**sST2 (ng/mL)**				
≥70.9	3/37	1.74	10/37	6.15
<70.9	10/37	10.18	13/37	13.47
[a]**sLR11 (ng/mL)**				
≥15.2	8/41	5.34	14/41	9.78
<15.2	5/33	4.12	9/41	7.76

[a]Patients were dichotomized based on median values of sST2 (70.9 ng/mL) and sLR11 (15.2 ng/mL), respectively.

Abbreviations: MACCE, major adverse cardiac and cerebrovascular event; sLR11, soluble form of low-density lipoprotein receptor relative with 11 ligand-binding repeats; sST2, soluble form of suppression of tumorigenicity 2.

Table S2. Multivariable Cox proportional hazard models of sST2 and sLR11 for all-cause mortality and MACCEs with adjustment of dialysis adequacy

	**All-cause mortality**	**MACCEs**
	[a]Adjusted HR (95% CI)	[a]Adjusted HR (95% CI)
**sST2**		
[b]sST2 ≥70.9 ng/mL (vs. <70.9)	10.513 (1.987-55.623)	3.544 (1.204-10.433)
[c]sST2 ≥ cut-off (vs. <cut-off)	14.804 (2.381-92.050)	4.147 (1.365-12.601)
sST2 (per 1 SD increase)	1.957 (1.048-3.655)	1.516 (0.956-2.405)
**sLR11**		
[b]sLR11 ≥15.2 ng/mL (vs. <15.2)	1.028 (0.109-9.732)	0.747 (0.279-2.003)
[d]sLR11 ≥ cut-off (vs. <cut-off)	2.366 (0.280-19.992)	1.291 (0.480-3.472)
sLR11 (per 1 SD increase)	0.407 (0.043-3.846)	0.902 (0.558-1.460)

[a]Adjusted HR was calculated after adjustment of age, sex, PD duration, diabetes mellitus, history of cardiovascular disease, smoking status, body mass index, hemoglobin concentrations, and weekly total Kt/V urea.

[b]Patients were dichotomized based on median values of sST2 (70.9 ng/mL) and sLR11 (15.2 ng/mL), respectively.

[e]Patients were categorized into two groups based on the calculated cut-off of sST2 for all-cause mortality (75.8 ng/mL) and MACCEs (72.5 ng/mL).

[d]Patients were categorized into two groups based on the calculated cut-off of sLR11 (14.9 ng/mL) for all-cause mortality and MACCEs.

Abbreviations: CI, confidence interval; HR, hazard ratio; MACCE, major adverse cardiac and cerebrovascular event; PD, peritoneal dialysis; SD, standard deviation; sLR11, soluble form of low-density lipoprotein receptor relative with 11 ligand-binding repeats; sST2, soluble form of suppression of tumorigenicity 2.

Table S3. Hosmer-Lemeshow test for calibration of models

Models	Chi-square statistic	P
**All-cause mortality**		
Base model	11.225	0.19
Model 1	12.276	0.14
Model 2	12.188	0.14
Model 3	7.957	0.44
**MACCE**		
Base model	7.510	0.48
Model 1	6.445	0.60
Model 2	6.692	0.54
Model 3	10.245	0.25

Base model: adjusted for age, sex, PD duration, smoking, diabetes mellitus, cardiovascular disease, body mass index, and hemoglobin

Model 1: base model + sST2.

Model 2: base model + sLR11.

Model 3: base model + hs-CRP.

Abbreviations: hs-CRP, high-sensitivity C-reactive protein; PD, peritoneal dialysis; MACCE, major adverse cardiac and cerebrovascular event; sLR11, soluble form of low-density lipoprotein receptor relative with 11 ligand-binding repeats; sST2, soluble form of suppression of tumorigenicity 2.

Table S4. Baseline characteristics of patients with and without sST2 and sLR11 values

	Participants with sST2 and sLR11 (*N* = 74)	Participants without sST2 and sLR11 (*N* = 29)	*p*
Age (years)	53.9 ± 11.8	54.8 ± 11.1	0.73
Male, *n* (%)	47 (63.5)	14 (48.3)	0.19
Duration of PD (months)	30.0 (16.0-96.0)	15.0 (9.0-38.5)	0.02
Diabetes mellitus, *n* (%)	21 (28.4)	7 (24.1)	0.81
Cardiovascular disease, *n* (%)	10 (13.5)	5 (17.2)	0.76
Smokers, *n* (%)	29 (39.2)	10 (34.5)	0.66
Lipid-lowering therapy, *n* (%)	28 (37.8)	11 (37.9)	0.9
SBP (mmHg)	133.7 ± 21.5	134.4 ± 23.7	0.30
DBP (mmHg)	75.0 ± 11.6	80.6 ± 13.2	0.77
BMI (kg/m^2)	23.1 ± 2.8	22.5 ± 2.8	0.78
Hemoglobin (g/L)	104 ± 11	107 ± 17	0.01
Glucose (mmol/L)	5.3 ± 2.4	5.5 ± 1.5	0.9
Blood urea nitrogen (mmol/L)	7.4 ± 2.1	6.8 ± 2.0	0.53
Creatinine (μmol/L)	972.4 ± 371.3	822.1 ± 291.7	0.26
Albumin (g/L)	35 ± 5	36 ± 4	026
Triglyceride (mmol/L)	1.3 ± 0.8	1.8 ± 1.1	0.05
Total cholesterol (mmol/L)	4.3 ± 0.9	4.6 ± 1.2	0.36
LDL cholesterol (mmol/L)	2.4 ± 0.7	2.4 ± 0.8	0.70
HDL cholesterol (mmol/L)	1.1 ± 0.3	1.1 ± 0.3	0.9

Calcium (mmol/L)	2.2 ± 0.2	2.1± 0.2	0.89
Phosphorous (mmol/L)	1.7 ± 0.5	1.6 ± 0.4	0.16
hs-CRP (mg/L)	0.96 (0.62-2.10)	1.19 (0.60-2.45)	0.63
Total Kt/V urea (per week)	2.1 ± 0.4	2.1 ± 0.4	0.9
nPCR (g/kg/day)	0.97 ± 0.17	1.0 ± 0.2	0.04

Note: Data are expressed as mean ± standard deviation, median (interquartile range), or number of patients (percent). Mann-Whitney *U*-tests were used for continuous variables and chi square tests were used for categorical variables.

Abbreviations: BMI, body mass index; BUN, blood urea nitrogen; DBP, diastolic blood pressure; hs-CRP, high-sensitivity C-reactive protein; HDL, high-density lipoprotein; Kt/V urea, fractional urea clearance; LDL; low-density lipoprotein; nPCR, normalized protein catabolic rate; PD, peritoneal dialysis; SBP, systolic blood pressure; sLR11, soluble form of low-density lipoprotein receptor relative with 11 ligand-binding repeats; sST2, soluble form of suppression of tumorigenicity 2.
